# From Friends across the Sea

**Published:** 1887-11-26

**Authors:** 


### FROM FRIENDS ACROSS THE SEA.

### BY OUR READERS' RELATIVES AND COLONIAL COUSINS.

THE letter we publish this week was written by a young man who is a dealer in gems. His usual residence is in Calcutta, but having gone "up country" to show some jewels to a Rajah who, it was hoped would make a large purchase, he narrates to the folks at home the incidents of the journey.

**A Journey from Calcutta "Up-country."**

On Thursday, the 25th of January, we crossed the river by steamer to the railway station, from which we started about nine p.m. The first night was most awfully cold. I could not keep myself warm at all. The cold here is very icy, though the thermometer is between 50 and 60 degs. I think the reason is that the air is clearer and thinner than in England. The only pretty part of the country is within 100 miles of Calcutta; so we missed seeing it, as we passed through in the night. After leaving Dinapore it is very flat and uninteresting, though now and then we could see a herd of deer, wild pig, and jackals, as well as large birds—cranes, &c. There were a great many pretty birds sitting on the telegraph wires.

**A New Method of Transit.**

We got out of the train at Kurtipore about eleven a.m. on Sunday, where we found elephants waiting for us, which we immediately mounted, and off we started for K——. While on the elephant I thought of the struggle we used to have for a ride at the Zoo in our childish days. There was no proper road made, so we went through fields *not* verdant—nothing but sand, and no end of rat-holes. The place looked something like a rabbit warren. The rats there are not the ordinary species, but are of a yellowish tint, and for all I could see they must live on air, for there was no vegetation. We arrived at K—— about two p.m., passed through the Bazar, or "poor town," where we saw some "chetahs" (a sort of leopard used for hunting) belonging to the Maharajah. Each one had a man to look after him—brush the flies off, and, if hot, fan him—the luxurious beast! When one man goes away another takes his place, for if all this were not done the animal might get savage and lose his taste for civilisation.

**An Uncomfortable Domicile.**

We stopped in front of a large brick building. On going in, we found the place very dirty and the windows broken, no furniture, etc. The house had been built and used by the English for military purposes some years ago, and had been allowed to get out of repair since. We asked our guides what they meant by bringing us here. They said that the "head man" had told them to do so, and he acts really for the Rajah. One of the late Rajahs was a great friend to Government, and did a great deal of service at the time of the mutiny. He married an Englishwoman, became a Christian, and commenced building a church; but his wife deserted him, whereupon he stopped building the church and commanded his people to turn back to their old religion. While in the town we came across a man who, when in Calcutta, had been "bearer" to a friend who was with me. This was very fortunate, as he was acting in the same capacity to the "head man" here; so he took us to a house, where we found a fine tent pitched in the garden, and every convenience, all at our service. We dined at seven p.m., this being the first food we had touched since leaving Calcutta.

**The Rajah's Palace.**

We went to the Palace in the evening. I cannot give you a very good idea of the building. I dare say you could find a better in the "Illustrated." It stands in a very large garden, and the building is surrounded or enclosed by a wall, which forms a sort of terrace all round the palace. You ascend a fine flight of stone steps leading into a very large lofty room, the walls of which are hung with large mirrors; chandeliers hang from the ceiling, and there is everything you might see in a European house, except a table; that, for some reason or other, is dispensed with. We had waited for some time before the Rajah appeared. A number of his Court were present, some of whom looked very imposing in their fine clothes and turbans.

**Princely Consideration**

We showed him our diamonds, and I saw at once that he would not buy anything. He said we had nothing good enough. It is, however, the custom of the native princes always to take something from the merchants, as the expense of the journey on purpose to show their goods is great. We reckoned the cost of ours at £50, and as the Rajah bought a ring and a gem, the profit on which about paid our expenses, I considered that on the whole the journey was satisfactory. We got back to Kurtipore just in time to see the train leave the station, so we had to put up at the bungalow. These bungalows exist all over the country on purpose for the convenience of European travellers. Fortunately, there was a goods train leaving at seven p.m., so we went in the guard's van as far as Kunnah, where we arrived about ten minutes to six a.m. I saw the sun rise over the Himalayas, and as each snowy peak showed up under the morning rays it had a very pretty effect. At Kunnah we succeeded in getting six coolies and two small ponies about ten o'clock, and then started for Nabbah.

